# Supplementary material for: Identification of dynamical changes of rabies transmission under quarantine: Community-based measures towards rabies elimination
Source: PLoS Comput Biol. 2023 Dec 15;19(12):e1011187. doi: 10.1371/journal.pcbi.1011187 (PMC10756519; doi:10.1371/journal.pcbi.1011187)
Supplement: Attachment — Submitted filename: PLOS_Response.docx [file pcbi.1011187.s001.docx]

**Resubmission of Manuscript**

Dear Editors,

Thank you for the opportunity to resubmit a revised version of our manuscript entitled "I*dentification of dynamical changes of rabies transmission under quarantine: community-based measures towards rabies elimination*”. We have carefully considered the reviewers’ comments and suggestions. Our point-by-point response is provided below (in italics) with our accordingly edited manuscript attached (please see highlighted changes) including modified Figures 4-7 and Tables 1-2.

We would like to thank reviewers 1 and 3 for their insightful comments and constructive feedback which we believe has significantly strengthened this paper. However, having read the review from reviewer 2, it is clear that they have completely misunderstood many aspects of the paper and their negative appraisal of our review is representative of this. There are three specific points that reviewer 2 has raised that we feel require a clarification in this response to the editors to ensure that a lack of understanding on behalf of reviewer 2 does not have negative implications for our paper. With this in mind, the three points that reviewer 2 raises are as follows:

1) The reviewer comments that it would be impossible to quarantine 45% of exposed dogs and that the authors should demonstrate that this is feasible. We believe that the reviewer has misunderstood the research that we present in our paper. We firstly present the results of a stability analysis of a deterministic model where we demonstrate that, in the absence of vaccination, 45% of infectious dogs would need to be quarantined for the disease free equilibrium to be stable. However, this is a theoretical analysis that, as stated within the paper, omits the stochasticity that is inherent in rabies transmission but in itself provides interesting insights into the levels of quarantine and vaccination that would be required in order for elimination to be achieved. Additionally, this theoretical analysis reinforces the fact that quarantine is insufficient to achieve rabies elimination without an active vaccination programme. We therefore next present the results from a computational model whereby quarantining of dogs is done through triage of patients who present at animal bite treatment centers, for which the parameters are informed by our field studies in the Philippines (Rysava et al. 2022). Thus, in this computational model dogs are only quarantined should they be identified through contact tracing and the resultant proportion of dogs that are quarantined is significantly lower than the 45% of the entire infected population that the reviewer claims. We are providing results from a computational model that simulates feasible quarantine practice that could take place through standard integrated bite case management protocols.
2) The reviewer claims that we are incorrectly using a 14-day quarantine period in our model, when a 10 day quarantine period is the WHO recommendation. They further go on to say that if our goal is to identify ALL exposed dogs then we assume a 4-month quarantine period in our model. Again, the reviewer has completely misunderstood our research. The purpose of our computational model is to impose quarantine for biting dogs that are identified through triage of patients at animal bite treatment clinics. We are not intending to propose protocols for quarantining all exposed dogs (and as stated above, the theoretical model investigates the level of quarantine that would be required for infectious dogs for given vaccination rates) but rather to investigate the impact of integrated bite case management that is focused upon quarantining of already identified biting dogs. Our model utilises data from the Philippines to parameterise the quarantine rates (Rysava et al. 2022) and in the Philippines a 14-day quarantine period for suspect dogs is generally implemented (Rysava et al. 2019; Rysava et al. 2022) and is recommended by the Philippines Department of Health and Bureau of Animal Industry (see <https://www.bai.gov.ph/media/5sbf3vzn/10-animal-quarantine-rabies-exposed-animals.pdf>, <https://rabies.doh.gov.ph/index.php/resources/faq#:~:text=The%20observation%20period%20for%20dogs,animal%20has%20bitten%20a%20person> and https://www.psmid.org/wp-content/uploads/2020/03/CPG-rabies-AO-2018-0013.pdf) . Our

model is therefore accurately capturing current practices for dog quarantine following bite incidents in the Philippines. The reviewer is therefore wrong to state that we are “incorrectly defining quarantine” in our model.

3) The reviewer challenges the author’s statements regarding the fact that rabies transmission between dogs occurs independently of population density and argues that there is substantial evidence that rabies is density dependent. We strongly dispute this - there is a significant body of work that provides evidence that frequency dependence influences rabies dynamics (e.g. ([Fitzpatrick et al., 2012](https://www.sciencedirect.com/science/article/pii/B9780128187050000200#bb0110); [Hampson et al., 2009](https://www.sciencedirect.com/science/article/pii/B9780128187050000200#bb0165); [Tian et al., 2018](https://www.sciencedirect.com/science/article/pii/B9780128187050000200#bb0340); [Townsend et al., 2013](https://www.sciencedirect.com/science/article/pii/B9780128187050000200#bb0355)) and that dog culling does not typically reduce rabies prevalence in practice (Townsend et al. 2013; Purwo Suseno et al. 2019), which would be expected if population density influenced dog biting behaviour. For rabies to exist at relatively low prevalence, a more nuanced frequency dependent approach is adopted here and is in line with previous work (see Rajeev, Metcalf and Hampson 2020 for a detailed explanation).

We have addressed each of these points (which the reviewer contests multiple times within their review) within the response but we felt it was sufficiently important to also address these in our response to the editors. Given the changes we have made to the manuscript and the response to reviewers that we have compiled, we hope that you will find our resubmission appropriate for publication.

Yours sincerely,

Kristyna Rysava

Michael Tildesley

**Reviewer #1**

In this study, Rysava and Tildesley develop a deterministic and stochastic modeling framework to study the effect of vaccination and quarantine in the context of rabies, a low prevalence endemic disease. The text is well written and the topic is of obvious importance. However, I feel that the analysis falls short in several respects, and would suggest that it be expanded upon prior to publication. My specific points are as follows:

**Larger points**
Particularly your stochastic model provides a powerful tool for looking at probabilistic events, and yet it doesn’t feel that this is taken advantage of. You refer repeatedly to the idea of population incursions from other populations vs “endogenous” ones arising from the long incubation period that may outlast the quarantine period, but you don’t explicitly characterize this. I would reformulate Figures 5, 6, and Table 2 in order to see how you can really take advantage of your stochastic model. Can you vary the population size (dogs and humans)?

For instance, right now when you report “number of infections” as in Figure 4, it’s not a very meaningful value since the size of the population is not contextualized. Maybe you can show how much disease introduction needs to occur from outside the population as a function of population size, given the possibility of these “endogenous” infections from exposed but not yet infectious individuals?

***Many thanks for the helpful feedback. We agree that the impact of incursions was not fully explored. We have now expanded our analysis by varying the incursion rate and run each simulation for 10 instead of 5 years to observe the longer-term changes. These additions are summarized throughout the Methods, Results and Discussion sections. We have also added a new figure to demonstrate case incidence over the modelled 10 years under increasing vaccination coverage and incursion rate values. We show that in the absence of exogenous incursions rabies fails to persist in the modelled population regardless of the vaccination rates. As such, we believe that endogenous incursions via leaky quarantine and possibly via dispersion around the R_0_ means do not play an important role in this model and we have clarified that in the text. Having said that, we agree with the reviewer that this is a relevant future direction to be explored with a more suitable framework capable of capturing the spatial distribution of susceptible individuals (arising from patchy vaccination efforts) which is likely to be more sensitive to such endogenous probabilistic processes. As for the used dog population, the model was partly parametrized on an existing population (described in Rysava et al., 2022). For those reasons we choose not to change the population size, but to make the values more meaningful we now report the infection as incidence per 10,000 rather than total counts.***

I think details of the stochastic model are missing. Can you explain more about how you implement the contact tracing for bites and the subsequent decision to quarantine and observe for symptoms? I know you describe the “overall” quarantine approach by scenario but I think more information is needed.

***We apologize for the lack of detail. We have now added more information on the motivation for quarantine scenarios based on a previously published study (Rysava et al., 2022) used to inform the stochastic model which also offers a focused description of how information on bite histories was collected and field investigations were conducted.***

I’m having a hard time understanding Table 2. Are these fits or just scenarios you’ve constructed with chosen values? First of all, I thought scenario 3 was a stricter quarantine scenario than scenario 2, yet all values for quarantine rates are lower? Why do the vaccination rates in dogs and humans systematically decrease with increasing R0? And what does “Quarantine incident rate (humans)” mean? Is that the incidence of infection in humans? I didn’t think humans underwent quarantine in this model. You say that Table 2 lets you see the effect of quarantine and vaccination rate, like Figure 5, but I don’t find that obvious at all. All the rates seem to co-vary, and I also don’t know what “incident rate” for quarantine or vaccination means.

***We apologize for the lack of clarity regarding table 2. We hope the table and the table caption are now improved. The incident rates are exponentiated coefficients of the presented explanatory variables taken from a negative binomial general linear regression model fitted to simulated time series data across a range of R_0_ and vaccination coverage values, and for the three quarantine scenarios. The incident rates have a multiplicative effect on the unit change in the response variable. For example, for R_0_=1.3, the expected number of canine rabies cases is 0.79 times the reference value (intercept, now added to the table) when scenario 2 is implemented as opposed to no quarantine, and 0.59 times the reference value when scenario 3 is implemented compared to no quarantine (i.e., reducing the expected mean of 55.87 to 44.58 and 33.13 for scenarios 2 and 3 respectively). The vaccination coverage is a continuous variable, so the change (incident rate) in the expected mean of canine cases is calculated for a unit increase in the vaccination coverage (1%) which is why it appears low - assuming the same R_0_, 50% vaccination coverage will bring the expected mean of 55.87 to 25.23.***

**Smaller points / questions**
I think it would help to justify the use of many different distributions for parameters, i.e. truncated normal for weekly proportion of bite-injury patients, quarantined dogs (Conway-maxwell-poisson), etc…

***We have now added several sentences throughout the computational methods section to clarify our choice of distributions.***

Your figures tend to be very hard to read (axis text and labels too small). In Figure 4, you could also add a label to the color bar for ease of understanding. Figure 5 has the same problems with readability as Figure 4. In some of the smaller plots, the y axis scale makes it such that it’s very hard to get anything quantitative out of the values for quarantine scenarios 2 and 3. In general I think some time could be spent improving their appearance.

***We apologize for the poor quality of our figures (they appeared fine as individual pdfs, but we should have checked more thoroughly when incorporated in the text). We have now edited the figures for readability.***

In Figure 4, providing “number of infected dogs” feels a bit out of context, since we don’t know anything about the population size we are working with. For instance you say >2100 infected dogs is more than empirical evidence, but for what size of population? Also the scale bar looks like the value in the bottom left corner of Figure 4B is closer to 3000?

***Thank you for pointing this out. Throughout the text in all the relevant figures, we have swapped the total numbers for incidence per 10,000 dogs to provide more contact to the actual scale of the infection.***

**Reviewer #2**

The authors describe a new method to model potential interventions to reduce rabies transmission in communities. The paper is largely well-written and the topic is of interest to the rabies modeling community. However, there are several critical issues with the methods and interpretations that would likely take significant effort to address (see below for more detail). Furthermore, the paper is not very helpful to rabies program managers without consideration to the feasibility of implementing quarantine programs to the levels that this analysis claims would be impactful. An accompanying analysis (even simple) of the cost and human resources necessary to operate a program that find 45% of exposed dogs (impossible, in my opinion) is necessary to bring this manuscript out of the realm of theory and actually show prospect for field-level impact.

***We would like to thank the reviewer for their comments on this manuscript. However, having read the review we feel that the reviewer has misunderstood key aspects of our analysis that may have affected their appraisal of this paper. We have addressed specific concerns below, but we note that our model does not recommend a programme to quarantine 45% of exposed dogs. In the first section of our paper, we present a theoretical model which demonstrates that, in the absence of vaccination, 45% of infectious dogs would need to be quarantined for elimination to occur. This theoretical model highlights that elimination is infeasible with quarantine alone and an active vaccination programme is required.***

***In the second section, we present results from a computational model in which dogs are identified for quarantine through triage of bite patients at animal bite treatment clinics. For this model, dogs are only quarantined from bites that have resulted in patients attending clinics and therefore the number of dogs entering quarantine in this model is significantly lower than the 45% posited by the reviewer. Our computational model has been developed and parameterised to capture realistic levels of dog quarantine and mirrors that which has been implemented at the animal bite treatment centers in our field study (see Rysava et al. 2022). We have clarified this within the theoretical model results section of the manuscript to make it clear to the reader that we are not advocating a policy where 45% of infectious dogs would be quarantined, which we agree is infeasible.***

**MAJOR CONCERNS:**
1) the authors are incorrectly defining "quarantine" in this model. There are no recommendations for a 14-day quarantine period. There is a 10-day post-bite observation period that is recommended by WHO and the purpose of this is only intended to assess if the biting dog (or cat) was infectious at the time it exposed a human. Dogs that are unvaccinated and exposed to another rabid dog are recommended to undergo a 4-month quarantine period, which is reflective of the rather long and variable incubation period in dogs. More details on this concern can be found below.

***The computational model presented in this paper investigates the impact of quarantine based upon triage of bite patients who present at animal bite treatment clinics and, as stated in the manuscript, is informed by a field project in the Philippines (Rysava et al. 2022). Therefore the suggested 4 month quarantine period is irrelevant to this study, given the focus on suspect dogs. Furthermore, in the Philippines the current recommendations are for a 14 day rather than a 10 day quarantine period for rabies-suspect dogs, as defined by the Bureau of Animal Industry and the Department of Health (see*** [***https://www.bai.gov.ph/media/5sbf3vzn/10-animal-quarantine-rabies-exposed-animals.pdf***](https://www.bai.gov.ph/media/5sbf3vzn/10-animal-quarantine-rabies-exposed-animals.pdf)***,*** [***https://rabies.doh.gov.ph/index.php/resources/faq#:~:text=The%20observation%20period%20for%20dogs,animal%20has%20bitten%20a%20person***](https://rabies.doh.gov.ph/index.php/resources/faq#:~:text=The%20observation%20period%20for%20dogs,animal%20has%20bitten%20a%20person)***,*** [***https://www.psmid.org/wp-content/uploads/2020/03/CPG-rabies-AO-2018-0013.pdf***](https://www.psmid.org/wp-content/uploads/2020/03/CPG-rabies-AO-2018-0013.pdf)***, Rysava et al. 2018 and Rysava et al. 2022). Therefore our 14 day quarantine period is correct in the model given the motivation for this work. We have clarified this in the manuscript.***

2) the authors cite 3 papers to make the bold statement that dog-mediated rabies is NOT density dependent. It should be noted that some of the most commonly cited and respected models in the rabies community have inherent density-dependent assumptions, including Coleman and Dye, which gives us the oft-cited "70%" recommendation and preceding transmission models by Zinsstag and Borse. The idea that rabies is NOT density dependent stems from several heavily biased and misinformed studies and is in direct conflict with all empirical evidence of rabies reductions after implementation of dog population management programs (e.g. Human Society programs to remove unwanted free-roaming dogs from communities). Furthermore, there are numerous wildlife rabies studies demonstrating population density dependence for transmission; it is difficult to believe that such a conserved viral genome would have no density dependence in free roaming dogs. The misunderstanding of density dependence and rabies stems from poor definitions of the susceptible dog population (clearly defined by Coleman and Dye) - when we only consider the dogs that are able to roam freely (at least part of the time) and become expose to rabid dogs, then there is strong evidence for density dependent transmission, as we see in wildlife systems as well.

***Whether rabies transmission depends on frequency or density is a very complex question and in fact neither is entirely true for such a strongly spatially structured disease. Whilst rabies may be density-dependent at a very small scale (<1 km^2), it does not apply to the population level as contact is limited by the spatial scale of host’s movement and in practice density-dependent models will fail to recuperate the observed trajectories. The assumptions in this paper build upon a significant body of work that provides supporting evidence that biting behaviour of rabid dogs is not, in fact, density dependence and that assuming frequency dependence for modelling purposes is more appropriate given the nature of transmission characteristics (***[***Fitzpatrick et al., 2012***](https://www.sciencedirect.com/science/article/pii/B9780128187050000200#bb0110)***;*** [***Hampson et al., 2009***](https://www.sciencedirect.com/science/article/pii/B9780128187050000200#bb0165)***;*** [***Tian et al., 2018***](https://www.sciencedirect.com/science/article/pii/B9780128187050000200#bb0340)***;*** [***Townsend et al., 2013***](https://www.sciencedirect.com/science/article/pii/B9780128187050000200#bb0355)***). Around the world, rabies typically exists in populations at relatively low prevalence regardless of the size of the population (***[***https://journals.plos.org/plosbiology/article/figure?id=10.1371/journal.pbio.1000053.t002***](https://journals.plos.org/plosbiology/article/figure?id=10.1371/journal.pbio.1000053.t002)***) and dog culling often does not reduce that prevalence (and certainly not R0), which strongly suggests that density dependence is not an appropriate assumption (Townsend et al. 2013, Porwo Suseno et al. 2019). We are a little confused by the reviewer’s comments that many papers that assume frequency dependence are “heavily biased and misinformed” since the reviewer has not provided any evidence to back up this damning claim. The papers that our research builds upon have been developed by world leading rabies experts, have been published in high profile peer reviewed journals, are highly cited and we are not aware of the criticism that the reviewer alludes to. We also note that dogs are not wildlife and exhibit substantially different social behaviour that is intrinsically linked to the human population, so it is inappropriate to use evidence from wildlife populations to justify density dependence for rabies transmission in dogs. To reassure the reviewer, we have expanded this section of the discussion to include additional references that support our model assumptions.***

3) As mentioned above, the lack of consideration to the logistical needs to implement the high level of quarantine identified in this model makes any real-world implications difficult to see happen.

***As stated above, we are not proposing that high levels of quarantine (e.g. 45% of infectious dogs) should be implemented in practice and have clarified this in the manuscript.***

4) The authors should be more clear about their assumption as to how these exposed dogs will be identified. The vast majority of rabid dogs will not bite people, therefore would not be identified in a bite-based triage system as described in this manuscript. So there are 2 populations of dogs that need to be considered: 1) dogs that get rabies and do NOT bite people and 2) dogs that get rabies and bite people (or are part of that traceback program). When the authors refer to 45% quarantine rates, which dog population are they referring to?

***As stated above, we are not proposing that 45% of all dogs should be quarantined. The theoretical model simply demonstrates the levels of quarantine that would be required for different levels of vaccination. The computational model is parameterised based upon triage of patients at animal bite treatment centers. We agree with the reviewer that many rabid dogs will not bite people and will not be identified through triage which is why in our model we are only considering those dogs that CAN be identified and quarantined through this triage process.***

**SPECIFIC COMMENTS:**
INTRO
Line 10: Incomplete sentence ending in “… by;”

***We have corrected this in the manuscript.***

METHODS
There is a critical flaw in the logic of this model. The recommended quarantine time for an EXPOSED dog is 4 – 6 months, to ensure that the dog does not develop rabies. The 14-day quarantine (which is actually 10 days, per WHO recommendations) is only to determine if a BITING dog is currently infectious with rabies virus and informs PEP decisions for the exposed PEOPLE. If the goal of this model is to identify free roaming dogs that are exposed to rabies virus, then I highly doubt the conclusions will be relevant, as these exposures typically go unrecognized by dog owners and community members. However, per the methods described, if the intention is to assume these exposed dogs CAN be identified, then the appropriate parameter value should be 4 to 6 months. <https://www.cdc.gov/rabies/specific_groups/veterinarians/potential_exposure.html#:~:text=If%20the%20owner%20is%20unwilling,or%206%20>(ferrets)%20months.

***As discussed above, our paper implements quarantine based upon triage of bite patients at animal bite treatment centers in the Philippines and therefore the assumption of a 14 day quarantine period is correct. We would also like to stress that the study system we cover in this manuscript is not ferrets in the US.***

Line 114: This sentence could be misleading when describing “n”. Finding dogs exposed after a HUMAN exposure is identified is fairly straightforward. However, many rabid dogs never bite a person and many bite victims never seek medical care necessary to initiate the triage process. Therefore, “n” as described here is only representative of a small proportion of the dogs that drive enzootic transmission.

***We agree that many rabid dogs may never bite a person and many victims may not seek medical care. We have therefore clarified this in the manuscript with the following sentence: “It is important to note that 𝜼 only captures those dogs identified through following rabid animals responsible for case patients and therefore is only a fraction of the total dogs that may be exposed or infected in the population at any time.”***

Line 114: Please provide citations for this “field observations” comment. If this refers to the studies from pastoral Tanzania, numerous other literature has been published in the last decade that conflicts with the observations from this study area, and likely the observations here are non-generalizable.

***This is not based upon pastoral Tanzania, but rather from research carried out by the authors in the Philippines and subsequently published (Rysava et al. 2022) hence is relevant for the model that is developed here. We have added this citation into the manuscript to clarify this. We do also note within the manuscript in this same paragraph that we take a conservative assumption for the value of 𝜂 and that further investigations are necessary to provide more robust estimates for 𝜂 in the future.***

Line 114: I don’t see where the model accounts for the proportion of exposed dogs that are not involved in a human exposure (from field observation, this is the vast majority of dogs). Where does the model account for this major driving of transmission, which would never be visible to the triage system that this model relies upon for the quarantine process?

***In the computational model section in the methods, we explain that the triage system identifies infected dogs based upon bite patients presenting at clinics. However, as explained in the model, the number of bites occurring from an infected dog is drawn from a negative binomial distribution such that not all infected dogs will bite other dogs (or humans), whilst not all humans that are bitten will present at clinic and be administered PEP (as defined by the 𝜺 and 𝝁 parameters respectively). Exposed dogs that are not involved in human exposure are therefore explicitly accounted for in the model but for the avoidance of doubt, we have amended this section of the methods to explicitly stated this in the manuscript as follows: “The model structure has been developed such that we are able to capture not just rabid dogs that bite other dogs and humans, but also rabid dogs that do not bite as well as potentially exposed humans that do not attend a clinic or receive PEP, thus providing an accurate representation of canine rabies dynamics.”.***

Line 115: Critical flaw – 14-days is not the correct parameter value. Exposed dogs, which are unvaccinated, are recommended to be quarantined for 4 – 6 months, which is estimated to reflect >95% of the incubation period. 14-days (which is actually 10) is for biting dogs and is only to ensure they were not shedding virus at the time a person was exposed. This paper has some parameter values that the authors could consider: Negligible risk of rabies importation in dogs thirty days after demonstration of adequate serum antibody titer - PMC (nih.gov) – While the author’s rationale for 14 days is incorrect, this paper does provide support that the immediate vaccination and quarantine of exposed dogs would only require a 30-day quarantine period (rather than 4 months). While official recommendations are still 4 months, scientific evidence for 30-day post-exposure quarantine periods is growing.

***As explained above, 14 days is the correct parameter value given the context of the study.***

Line 116: please use consistent language when referring to EXPOSED vs CASE patients. I think in this sentence the authors mean “exposed”, rather than rabies cases.

***The text has been now edited to ensure consistency regarding patient status and introduced the terminology explicitly when first introduced. Thank you for pointing this out to us.***

RESULTS:
Line 120: Ro is almost certainly related to the density of SUSCEPTIBLE dogs in the community. Rather than randomly allowing the model to reflect a wide range of Ro, why not assume a level of density-dependence to reflect affected communities?

***As we have stated above, our research builds upon a significant body of work that provides substantial evidence to imply that R_0_ is not related to the density of susceptible dogs within the local community and hence we assume frequency dependent transmission. Several highly cited research papers providing support evidence for this assumption (***[***Fitzpatrick et al., 2012***](https://www.sciencedirect.com/science/article/pii/B9780128187050000200#bb0110)***;*** [***Hampson et al., 2009***](https://www.sciencedirect.com/science/article/pii/B9780128187050000200#bb0165)***;*** [***Tian et al., 2018***](https://www.sciencedirect.com/science/article/pii/B9780128187050000200#bb0340)***;*** [***Townsend et al., 2013***](https://www.sciencedirect.com/science/article/pii/B9780128187050000200#bb0355)***; Rajeev, Metcalf and Hampson 2020).***

Line 122: Please be more descriptive in talking about the quarantine rates. Are these the rates of ALL EXPOSED DOGS that would be quarantined (45% is unimaginably high), or is this the fraction of dogs that are identified through contact tracing that are found and placed into quarantine?

***We have responded to this above and clarified this in the manuscript.***

Line 123: the authors should be aware of several papers (Japan, notably) documenting Ro much higher than 2. However, I think a consideration for past and this current model is that Ro is not a stable value for rabies, and Rt is a more appropriate value to consider. Rt can vary drastically based on the stage of the epidemic and the density of susceptible dogs.

***We are unsure which papers the reviewer is referring to here. Rabies was eliminated in Japan in the 1950s, though there was some recent modelling work that utilised data from 1914-1933, obtaining a R_0_ value of 2.4 for the first wave and 1.5 for the second wave (Kurosawa et al. 2017). This may be the work that the reviewer is referring to, but R_0_ is not much higher than 2 here and we believe that this provides supporting evidence that our assumptions regarding R_0_ in our work are appropriate. Other research provides evidence for a relatively narrow range for R_0_ regardless of the population under consideration (Hampson et al. 2009, table 2). Regarding the use of R_0_, rather than R_t_,we believe that this is suitable metric to use. Of course as an epidemic progresses, the number of secondary infections that occur changes as a result of a depletion of susceptibles and an introduction of intervention measures (hence R_t_ is typically used to indicate infections that will occur in these conditions) yet R_0_ remains a suitable measure for our work in a low prevalence setting where density is not a driver of transmission - in our model we utilise the value of R_0_ to draw the number of infectious bites from a negative binomial distribution, which is used to capture the substantial variability in biting behaviour of infectious dogs. The infectious bites are then distributed in the population proportionally to the size of each compartment. We therefore believe that our modelling assumptions are appropriate.***

Paragraph 134: The manuscript is not very clear when it comes to explaining if the model is only accounting for quarantine of the proportion of dogs that are involved in a human bite incident, or if they consider all dogs that participate in enzootic transmission. A large proportion of dogs will develop rabies and never bite a person – they will drive transmission regardless of how robust the human bite triage system operates. What assumptions (and these would be critical) did the authors make in regards to the proportion of rabid dogs that actually bite people (human-dog transmission rate)?

***We have clarified our quarantine assumptions in the manuscript and have answered this concern in response to other reviewer’s comments above.***

A model that has been published several times accounts for this parameter and estimates this dog-to-human transmission rate at 0.00003 km3/dogs/week, and later updated to 2.34*10^-5 by Kunkel et. al. These values are not directly transferrable to the modeling method the authors have chosen, but they highlight the longstanding recognition that the majority of rabid dogs do not expose people. If human bites are the impetus for quarantine in this model, then it needs to better explain how it accounts for the proportion of dogs that do not expose people but drive enzootic transmission in a community.
<https://www.ncbi.nlm.nih.gov/pmc/articles/PMC5988334/#pntd.0006490.s001>
The urgency of resuming disrupted dog rabies vaccination campaigns: a modeling and cost-effectiveness analysis - PMC (nih.gov)

***This is an important piece of knowledge and deserves more work on its own, but it is not the scope of this paper. The model is not trying to address elimination efforts, and the authors stress the importance of mass dog vaccination if elimination is to be achieved throughout the manuscript.***

Paragraph 135: these findings are difficult to believe, given that the quarantine period selected for the model is incorrect. Furthermore, as mentioned, it needs to be more clear what total proportion of exposed dogs the authors are calculating.

***As has been mentioned above, our quarantine period is representative of that in place in the Philippines, so is correct given the scope of our work. We have also clarified above how quarantine is implemented in the model.***

Paragraph 138: this is just completely false. The 70% value determined by Coleman and Dye assumes density dependence. The three papers cited for this statement are well-known to be poorly collected data and at least one incorrectly defined susceptible dog populations. Perhaps the most well-cited rabies transmission model (Rabies econ) assumes density dependence, and defines SUSCEPTIBLE dogs as those that are able to roam freely at least part of the time. When models do not consider the confinement status of dogs, then density dependence associations become weak; this is a limitation of the assumptions in those models. The Morters paper has not stood up well over time, as there are many empirical examples where the increased confinement and reduction of dog populations through human measures has drastically reduced rabies cases.

***As we have stated above, we base our assumptions here on a significant body of work that provides evidence that rabies transmission is not density dependent except perhaps at a very small scale. We have expanded upon this in our response above. We are unsure why the reviewer has stated that “The Morters paper has not stood up well over time” since they have provided no evidence to substantiate this claim. This paper is in a leading peer-reviewed journal and has been cited over 200 times, so it is evident that this research is highly regarded by the scientific community.***

Overall: the authors should consider including an element of feasibility into the analysis; particularly feasibility of the health system resources to identify and quarantine 45% of ALL rabies exposed dogs… (impossible) and the cost to operate such a system. Would these all be in-home quarantines? If so, do we anticipate 100% owner compliance? Would these be at specialized facilities? If so, the cost alone would make this approach impossible for nearly all LMICs.

***As stated several times in our response, we are not considering 45% of all rabies exposed dogs in our model as being a feasible approach to take. Our computational model considers quarantine of dogs identified through triage of bite patients at animal bite treatment centres. We assume in this case that dogs would be quarantined at home, which is feasible given the relatively small number of dogs this would involve (much lower than the 45% of the population that the reviewer states). Our computational model assumes 100% compliance for all dogs identified for quarantine but accounts for the fact that exposed dogs may not show clinical signs during the 14-day observation period and will therefore become infectious following their exit from quarantine.***

**Reviewer #3**

I found this an interesting and mostly clear read, addressing the effectiveness of quarantine for rabies and rabies like diseases. I think this modelling work is useful and interesting. I have only relatively minor comments.

***We would like to thank the reviewer for their positive appraisal of our manuscript.***

Points to consider (line numbers are not working quite right so referring location as best I can)

L3: what is meant by ‘deceivingly easy’, that contact tracing is harder than it seems? Or not as effective as might be expected in leading to parameter estimates? Or something else?

***Both, to a certain extent. Unobserved events play an important role, especially for a low prevalence disease such as canine rabies. However, it is mostly that the traditional epidemiological models do not offer structures to capture the mechanistic processes of rabies transmission often leading to poor or unrealistic parameter estimates. We have now edited the paragraph providing more details.***

L7: ‘the system’ is a bit vague, what exactly is being referred to by this term?

***This has now been clarified in the manuscript.***

L32: ‘Both’? It isn’t clear what the two ‘things’ are?

***This has now been edited to clarify what is meant here.***

L35: ‘particularly effective for highly transmissible diseases’ – is this a quantifiable fact? It seems like this might be hard to establish.

***We agree with the reviewer. The sentence was not supposed to reflect on the actual effectiveness of vaccination to highly transmissible diseases, rather on the promising prospects of vaccination as a control tool to diseases that would otherwise lead to many individuals being infected. We have now reworded the sentence accordingly.***

L46: I’d think the reason quarantine is rarely applied for human to human transmitted diseases is that there are all sorts of issues with its enforcement and societal compliance, rather than a lack of evidence it's effective.

***Thank you for pointing this out, we agree and have reworded the sentence accordingly clarifying the difference in quarantine implications to human and animal populations.***

L51: perhaps ‘, we are specifically interested .. ‘

***Thank you for noting, we have edited the sentence now.***

L75: The delta terms would benefit from a bit more explanation. Perhaps comment on the assumptions underlying this formulation of density dependence?

***This formulation has been used elsewhere to simulate rabies dynamics (Hampson et al. 2007), such that density dependent deaths occur at a rate given by N*(b-d)/K, thus accounting for a population carrying capacity. We have clarified this in the manuscript and added this reference.***

L95: Perhaps indicate this is the Jacobian evaluated at the endemic equilibrium

***This has now been explicitly stated in this section of the paper.***

L96: Does the assumption that N is fixed equate to b = d? Indeed .. is N fixed as a result of the delta term even if b > d? How restrictive is the assumption that N is fixed?

***In the absence of infection in the population, the assumption that N is fixed would equate to a situation where birth rates are effectively equal to natural death rates. However, when rabies is spreading through the population, if we assume a density dependent carrying capacity then the assumption that N is fixed implies that birth rates will exceed natural death rates, given that there will be additional deaths associated with infection. We have clarified this in the manuscript.***

I found the description of the computational model unclear/confusing in a number of places. As I understand it, the human model is included only for the purposes of contact tracing, and the model considers PEP administration only as a by-product of attending a clinic and therefore becoming potentially involved in contact tracing? It feels a bit odd – given the way the model is described - that there is no discussion at all of the impacts of quarantine on human health outcomes although I recognize this could be regarded as out of scope. But if it is out of scope, perhaps focus more on the human dimension as necessary for the purposes of contact tracing, and not health seeking behaviour.

***The reviewer's understanding of the model is correct. Whilst quarantine as modelled here depends on the health-seeking behaviour of bite patients, there are also indirect impacts of quarantine on human health/exposed case incidence through the reduced number of rabid dogs circulating in the population. We have tried to demonstrate this in Table 5 which is now improved for clarity and elaborated in more detail in the Discussion section.***

There are rather a lot of ‘In fact’ and ‘As such’ phrases that seem to serve no purpose.

***Thank you for raising this issue. We have read through the manuscript and reduced the number of these phrases.***

Between L105 and L106: I’m not sure that it’s true that deterministic models cannot be applied to open population dynamics. Of course, it would require that flows ‘in’ and ‘out’ were modelled deterministically.

***We agree with the reviewer’s comment. The objective of this sentence was to note that deterministic models do not take into account stochastic events, and we have therefore deleted the section referring to closed populations such that the new sentence reads: “Stability analyses of deterministic models ignore the role of chance with regards to the pathogen extinction and reintroduction (both locally and globally) despite its influential impact on the future trajectories for diseases that operate at such low transmission levels.”***

Between L108 and L109: I’m confused by the negative binomial model here. Different k’s for humans and dogs? I’m not sure how this works. One NB distribution for dog bites and another for dog human bites? In which case what are the respective R0 values? Perhaps some more clarity here.

***We apologize for the lack of clarity. We have now added further explanation on how the transmission parameter k differs between dogs and humans, stressing that whilst the average number of exposures (R_0_) stays same for humans and dogs, the variance around the mean is narrower for humans.***

Between L110 and L111 : ‘The true rate at which a location receives an incursion’ … the use of location here risks confusion as this is not a spatial model as I understand it.

***We have now reworded the paragraph, talking about “populations” rather than “locations”.***

Between L112 and L113: the introduction of ‘case’ and ‘non-case’ incidents would benefit from more explanation.

***We have clarified the definitions of case and non-case patients in the text.***

Between L120 and L121: Presumably the destabilization of the endemic equilibrium point coincides with it ceasing to exist in the positive quadrant? I wonder if from an epidemiological point of view, focusing on I* might be more informative than its stability since the ‘return time’ to the endemic equilibrium is seemingly not of interest.

***The reviewer is correct that the destabilization of the endemic equilibrium is a result of the equilibrium point occurring for negative values of infected individuals. In the manuscript we had attempted to demonstrate this by the second set of panels in figure 4 where we plotted the values of E*+I* as quarantine percentage and vaccination rate vary. However, in line with the reviewer’s suggestion we now separately present values of E* and I* which highlights that the endemic equilibrium only exists for positive values of E* and I* with quarantine percentage and vaccination rate below certain values.***

Between L122 and L122: ‘However, to maintain disease endemicity for R0 = 1.3 in the absence of both interventions, the required number of Infected dogs (I∗) in the population reaches significantly higher values than what is suggested by empirical evidence (> 2110).’ I didn’t understand this remark. I think you are saying that when R0 = 1.3 and there are no control measures in place, the model predicts unrealistically high prevalences? Perhaps rephrase avoiding the use of ‘required’ and stating what the prevalence is since 2110 doesn’t convey this point so clearly. What is the carrying capacity (K) .. perhaps state this in the main text.

***We apologize for the poor wording in this instance; it has been brought up by reviewer 1 as well. We have now edited the results section and figures to conceptualize the infection in the population with respect to the population size rather than as total counts.***

Between L127 and L128: I’m curious as to exactly why the stochastic model makes different predictions about elimination. Is this because there is an incursion process in the stochastic model but not the deterministic model? (what happens if the incursion process is ‘turned off’ in the stochastic model?). Or the stochastic model isn’t run for long enough? Or is it simply the effect of variance around previously fixed means?

***This is an interesting question, thank you. There are three mechanisms that are likely contributing to the longer timelines until and higher thresholds for the elimination to be achieved. Incursions are certainly the most important one, and we have now expanded our analysis by testing different incursion rates. When there are no exogenous incursions entering the modelled population, rabies transmission appears to quickly die out. In addition, the stochastic model allows for leaky quarantine - exposed dogs that are temporarily quarantined but do not show any symptoms of the disease/won’t become infectious by the end of the quarantine period are returned to the population and can cause further infection despite their previous quarantine status. Lastly, the variance around the fixed R_0_ means may modulate the timelines until elimination, but it’s unlikely to be the main determinant in this model (non-spatial with fixed vaccination rate). We, however, hypothesize that this would be different in real life as the spatial distribution of susceptible individuals varies with patchy vaccination efforts.***

Between L132 and L133: It makes sense that contact tracing and quarantine are easier to implement when there are fewer cases. But in your study you use quarantine to address an endemic situation – albeit a low prevalence pathogen. Perhaps add this ‘third’ condition (endemic pathogens persisting at low prevalence)?

***We have now edited the sentence and mentioned an endemic scenario under which quarantine can be applied and useful to control pathogen spread.***

Fig 5 is painfully hard to see. Legend ‘range of R0 values’. Perhaps dispense with the ‘please’.

***We apologize for the poor resolution of the figures. The size of the legend and axes has been increased and the caption edited.***

Fig 6. I was puzzled by the seeming regularity in the amplitude of the oscillations. Is this a function of strict density dependence?

***We believe this pattern was caused by the steady rate of exogenous incursions that the disease transmission in the modelled population depended on. We have now tested varying levels of incursion rates to investigate this behaviour and demonstrated that in the absence of incursions rabies ceases to circulate in the population. We summarized this in a new Figure 6 and in parts of the Methods and Discussion sections.***

Add a statement indicating where the code can be found.

***We have now added a statement on Data availability. Both the code and with model variables and parameters can be found at our public Github repository https://github.com/IstyRsquared/Quarantine-Models.***

**References**

Rysava K, Espineda J, Silo EAV, Carino S, Aringo AM, Bernales RP, et al. (2022) One Health Surveillance for Rabies: A Case Study of Integrated Bite Case Management in Albay Province, Philippines. Frontiers in Tropical Diseases 3. doi:10.3389/fitd.2022.787524.

K. Rysava, M.E. Miranda, R. Zapatos, S. Lapiz, P. Rances, L.M. Miranda, M.C. Roces, J. Friar, S.E. Townsend, K. Hampson (2019). On the path to rabies elimination: The need for risk assessments to improve administration of post-exposure prophylaxis. Vaccine 37(1), A64-A72. <https://doi.org/10.1016/j.vaccine.2018.11.066>

Hampson, K, Dushoff, J, Bingham, J, Brückner, G, Ali, Y & Dobson, A (2007) Synchronous cycles of domestic dog rabies in sub-Saharan Africa and the impact of control efforts. Proceedings of the National Academy of Sciences 104 (18), 7717-7722. <https://doi.org/10.1073/pnas.0609122104>

Fitzpatrick, MC, Hampson, K, Cleaveland, S, Meyers, LA, Townsend, JP & Galvani, AP (2012) Potential for rabies control through dog vaccination in wildlife-abundant communities of Tanzania PLoS Neglected Tropical Diseases 6 (8), p. E1796. [10.1371/journal.pntd.0001796](https://doi.org/10.1371/journal.pntd.0001796)

Hampson, K, Dushoff, J, Cleaveland, S, Haydon, DT, Kaare, M, Packer, C *et al.* (2009)

Transmission dynamics and prospects for the elimination of canine rabies. PLoS Biology 7 (3), p. E53. [10.1371/journal.pbio.1000053](https://doi.org/10.1371/journal.pbio.1000053)

Tian, H, Feng, Y, Vrancken, B, Cazelles, B, Tan, H, Gil, M *et al.* (2018) Transmission dynamics of re-emerging rabies in domestic dogs of rural China. PLoS Pathogens, 14 (12), e1007392–18. [10.1371/journal.ppat.1007392](https://doi.org/10.1371/journal.ppat.1007392)

Townsend, SE, Sumantra, IP, Pudjiatmoko, Bagus, GN, Brum, E Cleaveland, S *et al.* (2013) Designing programs for eliminating canine rabies from islands: Bali, Indonesia as a case study. PLoS Neglected Tropical Diseases, 7 (8), p. e2372. [10.1371/journal.pntd.0002372](https://doi.org/10.1371/journal.pntd.0002372).

Purwo Suseno, P, Rysava, K, Brum, E, De Balogh, K, Ketut Diarmita, I, Fakhri Husein, W, McGrane, J, Sumping Tjatur Rasa, F, Schoonman, L, Crafter, S, Putu Sumantra, I & Hampson, K. (2019) Lessons for rabies control and elimination programmes: a decade of One Health experience from Bali, Indonesia. Rev Sci Tech. 38(1), 213-224. doi: 10.20506/rst.38.1.2954.

Rajeev, M, Metcalf, J and Hampson, K (2020) Chapter 20: Modeling rabies virus transmission dynamics. Rabies (Fourth Edition) Scientific Basis of the Disease and its Management, 655-670. <https://doi.org/10.1016/B978-0-12-818705-0.00020-0>

Kurosawa, A, Tojinbara, K, Kadowaki, H, Hampson, K, Yamada, A and Makita, K (2017). The rise and fall of rabies in Japan: A quantitative history of rabies epidemics in Osaka Prefecture, 1914-1933. PLoS Negl Trop Dis. 11(3), e0005435. doi: 10.1371/journal.pntd.0005435.
