# Supplementary material for: Identification of dynamical changes of rabies transmission under quarantine: Community-based measures towards rabies elimination
Source: PLoS Comput Biol. 2023 Dec 15;19(12):e1011187. doi: 10.1371/journal.pcbi.1011187 (PMC10756519; doi:10.1371/journal.pcbi.1011187)
Supplement: Attachment — Submitted filename: PLOS_Response2.docx [file pcbi.1011187.s002.docx]

**Resubmission of Manuscript**

Dear Editors and Reviewers,

Thank you again for the valuable feedback and the opportunity to share our work entitled "*Identification of dynamical changes of rabies transmission under quarantine: community-based measures towards rabies elimination*”. We have addressed all the remaining comments below on point-by-point basis (in bold italics) and edited our manuscript accordingly (highlighted changes).

Yours sincerely,

Kristyna Rysava

Michael Tildesley

**Reviewer #1**

Overall much improved and addresses my comments. I have a few minor questions remaining that it would be nice to address, but should not impact the decision for publication:

***Thank you.***

Text is much better in new Figure 4. The caption makes it seem like B and C should be comparable / same parameters, yet the R0 value used is different in the left panels of both – is this intentional? Also, it might be worth pointing out the different colormap scale. Right now if you looks quickly it seems like exposed incidence is lower than infection, but this is because the R0 is lower, and also it’s a different color scale.

***Thank you for pointing the inconsistency out – it was not intentional! We have now fixed the figure, so that all of the main panels refer to R0=1.3.***

***As for the colour map scale, we appreciate the reviewer’s concern and partially agree. However, when both infected and exposed incidence are put on the same scale (i.e., ranging from 0 to ~250), the infected incidence is illegible as all values are much lower than those of exposed incidence. We pasted the alternative figure for reference.***

Why do all the time series seem to just uniformly decrease? There doesn’t seem to be an “endemic” cycle (Figure 7). It seems like if you waited longer than 10 years, incidence may die out in all scenarios. I guess there are no seasonal drivers in the model, but I’m still a bit confused by this. Related, in Figure 6 when you plot monthly incidence by scenario, are you just plotting every single cumulative monthly value? So in reality is a range of numbers that decreases in time that you are plotting?

***In many parts of the world where rabies remains endemic, the disease circulates at a remarkably low prevalence, sustained though incursions from interconnected metapopulations. As such, the incidence is unlikely to die out except for the “no-incursion” scenarios. In fact, when there are no exogenous incursions entering the modelled population, rabies transmission fails to persist. We, however, appreciate, the prevalence reported from our model may differ from one emerging from more realistic settings given our model is non-spatial and set with fixed vaccination rate. We therefore hypothesize the prevalence, and overall dynamics of rabies would be modulated in real life by the spatial distribution of susceptible individuals which varies with patchy vaccination efforts. We discuss this limitation in the second to last paragraph of the Discussion section.***

***As for Figure 6 – we agree. The value of this figure, however, is in demonstrating the relative difference between the tested scenarios rather than in purely documenting the absolute amount of infection.***

In Table 2 caption, what is “unit increase in vaccination effort”? Does that mean a 1% increase in the vaccination rate?

***That’s correct and now clarified in the text of the table caption as well.***

**Reviewer #3**

I'm satisfied that my original points have been addressed. My only additional suggestion is that the authors provide a reference to the tau leap methodology they refer to.

***Thank you. We have now added appropriate references for the tau leap methodology in the text and here:***

Gillespie, Daniel T. 2001. Approximate accelerated stochastic simulation of chemically reacting systems. *J. Chem. Phys*. 115 (4).

Keeling, Matt J., and Pejman Rohani. Modeling infectious diseases in humans and animals. Princeton University Press, 2008.

Padgett, Jill M. A., Ilie, S. 2016. An adaptive tau-leaping method for stochastic simulations of reaction-diffusion systems. AIP Advances. 6 (3).
